# Adolescent mental health in post-conflict communities: results from a cross-sectional survey in Northern Uganda

**DOI:** 10.1186/s13031-023-00549-2

**Published:** 2023-11-02

**Authors:** Heather Wipfli, Kyra Guy, Abigail Kim, Peninah Tumuhimbise, Kenneth Odur, Lira Youth Public Health Ambassadors, Adiro Susan, Adupa Stephen, Akello Rebecca, Alum Nancy, Anyima Fredrick, Aoko Emily, Awello Monica, Ejang Winnie, Acio Barbara, Akao Winnie, Alum Recho, Angom Salume Precious, Aol Rachael, Awidi Fiona Tabitha, Ejang Brenda, Kia Judith, Lalita Ruth Amongi, Okello Moses, Olem Jasper, Adongo Marrion, Amono Monica, Awino Mirriam, Okello Denish, Okello Geoffrey Ocama, Obwona Jimmy, Akullu Christine

**Affiliations:** 1 USC Global Research, Implementation, and Training Lab, Department of Population and Public Health Sciences, University of Southern California, 3518 Trousdale Pkwy CPA 353, Los Angeles, CA 90089 USA; 2 Energy In Action, 941 Wiladonda Drive, La Canada, CA 91011 USA; 3 Children’s Chance International, Central Division, Lira Municipal Council, Plot 20 Otim Lakana Road, P.O. Box 147, Lira, Uganda; 4 International Relations/Political Science, 837 Downey Way, Los Angeles, CA 90089 USA

**Keywords:** Mental health, Uganda, Political determinants of health, Adolescents, Community-based participatory research

## Abstract

**Purpose:**

This study evaluated adolescents' mental health their knowledge, attitudes, and beliefs about mental health conditions, and their access to critical mental health services in Lira District, northern Uganda. The political history of the region, the epicenter of the decades-long conflict between the Lord’s Resistance Army and the Ugandan government, makes for an interesting case study of the political and social determinants of mental health of those directly impacted by conflict, and on subsequent generations growing up in post-conflict communities.

**Methods:**

This paper presents the results of a community-based participatory research study carried out by youth public health ambassadors in Lira District, Uganda. The study consisted of a mixed methods cross-sectional survey of households, schools, and healthcare facilities.

**Results:**

The study found 66% of adolescents indicated poor well-being and possible symptoms of depression and 41% of adolescents reported at least 4 childhood trauma events. Over 35% reported feeling extremely sad and 60% reported feeling socially isolated during the COVID lockdowns that lasted from 2020 to 2021. Nearly half of the adolescents aged 14–17 surveyed (N = 306) believed that witchcraft caused mental health problems, while less than 20% believed that traumatic experiences could be a cause. Forty percent of respondents had no idea of where to seek mental health care, and few facilities had mental health services available.

**Discussion:**

These findings illustrate the need to study the political and social determinants of mental health, especially on those directly impacted by armed conflict and for the generations growing up in post-conflict communities as they seek to rebuild.

## Introduction

According to the World Health Organization (WHO), mental health disorders currently affect more than one billion people, including one in seven adolescents [1]. Already a leading cause of death and disease, the COVID-19 pandemic led to a stark rise in the number of mental disorders [2]. Anxiety and major depressive disorders and mental health conditions alone cost the global economy over US$ 1 trillion each year [3]. However, despite the growing health and economic burden, governments currently spend approximately 2% of their health budgets on these disorders and people with mental health conditions often experience severe human rights violations, discrimination, and stigma [4].

In response to the call for greater attention to mental health challenges in low-income and post-conflict communities, a community-based participatory research (CBPR) study was carried out in Lira District, Uganda in the Spring of 2022. The study aimed to evaluate the mental health of adolescents (14–17 years of age), along with their knowledge, attitudes, and beliefs about mental health conditions and their access to critical mental health services. The political history of Lira District, the epicenter of the decades-long conflict between the Lord’s Resistance Army (LRA) and the Ugandan government, makes for an especially interesting case study of the political and social determinants of mental health of those directly impacted by conflict, and on subsequent generations growing up in post-conflict communities. At the height of the conflict, which lasted from 1996 to 2006, 1.7 million people lived in squalid displacement camps across the region [5]. The LRA was known to be particularly brutal against children, kidnapping thousands of them to serve as child soldiers. While mental health risks can manifest themselves at all stages of life, those that occur during early childhood are particularly detrimental [6]. This paper presents the results of the study and discusses how to address some persistent barriers and advocate for better, mental health care in the region and around the world.

## Methods

The study consisted of a mixed methods cross-sectional survey of households, schools, and healthcare facilities. The study was approved by the Uganda National Council for Research and Technology (GUREC-2021-84), and the University of Southern California (USC) (UP-23-00478) internal review board.

### CBPR process

Data was collected by 30 community-based Youth Public Health Ambassadors (YPHAs) aged 17–22 who were recruited and trained in public health practices and data collection from the three sub-counties (Agweng, Ayami, and Aromo) in Lira District. YPHA recruitment requirements included age, availability, ability to read and write in both English and Lango languages, willingness to volunteer, and commitment to community work. The process of recruiting the youth included reaching out to local council representatives, parents, and youth to explain the study, its objectives, and the selection process. To encourage female empowerment in the community, more girls were recruited for the YPHA program than boys.

After selection, the youth ambassadors received basic public health education through a series of workshops. The first workshop, held in February 2021, included interactive lessons on vector-borne disease, sanitation and hygiene, sexual and reproductive health, risky behaviors, and mental health. At the end of the workshop, the YPHAs participated in a DELPHI exercise to identify public health issues in their communities through which mental health was prioritized [7]. A second workshop, held in April 2021, focused on the development of a community-wide health assessment utilizing CBPR methods which engage community members as active and equal partners in research and allows them to investigate issues of concern in their own communities [8].

### Study sites and participants

YPHAs collected data from households and adolescents living in the homes, schools, and health care facilities within six parishes located in three sub-counties within the Lira District of Uganda.

#### Households/adolescents

Approximately 50 households in each sub-county with at least one adolescent between 14 and 17 years old were selected based on convenience. Household consent to participate in the study was granted by the head of the household, while the adolescents in the home also provided their consent to be surveyed. To promote open communication, adolescents were gender-matched with their YPHA interviewer and interviews took place out of earshot of adults in the household. Small incentives were provided to participating households including 1 package of either posho, rice, soap, mosquito nets, or reusable menstrual pad kits.

#### Schools and healthcare facilities

YPHAs identified public and private primary and secondary schools present in their Parish and approached them to obtain consent to participate in a facility observation and survey. Regarding healthcare facilities, at the district level in Uganda, the health service is administered through four levels of care or Health Centers I–IV. Ambassadors identified Health Centers II–IV (ranges from providing curative to basic preventive to outpatient care) present in their Parishes, as well as select specialty care centers, and Children’s Chance International (CCI) Uganda staff contacted them to receive consent to participate in the study. Small incentives were provided to participating schools and health facilities, including basic supplies that included 1 package of soap, hygiene kits, and masks each.

### Study design

Quantitative data regarding mental health status, knowledge, beliefs, and access to services were collected using survey and observation assessments at households, while surveys of infrastructure, resources, and programming and facility observations were carried out at schools and health care facilities. All data collection instruments were translated into Luo, the local language, by a qualified translator hired by CCI.

#### Household/adolescent data collection

At each house, the head of the household was surveyed about the basic household demographics, a household observation tool was completed, and an adolescent in the home was surveyed. The Adolescent questionnaire assessed adolescents’ general demographics, physical health status, diet, substance use, violence and risky behaviors. Mental health status and behaviors was assessed using the Adverse Childhood Experiences Score (ACE-IQ) and the WHO-5 Wellbeing index mental health knowledge.

#### School and health facility data collection

School and health facility questionnaires were carried out with the head of the school/facility assessing mental health services and support provided for adolescents, impacts of COVID-19, staffing and management, facilities infrastructure and technology, and communications with Village Health Teams. An observation tool was also administered to assess the state of the facilities.

### Data management, analysis, and dissemination

YPHA’s initially noted the responses to surveys and recorded observations on paper and later entered the responses into Qualtrics using handheld tablets preloaded with the survey instrument. Student researchers at the USC GRIT Lab carried out initial data analysis using STATA 17 for windows. Gender differences in adolescent self-reported health behaviors and health outcomes were analyzed using chi-square tests. Multivariate logistic regression was used to assess the association between adolescent WHO-5 index depressive symptoms, self-reported social support, and adverse childhood experience score categories. WHO-5 index depressive symptoms were determined by a WHO-5 well-being index score of  ≦ 28, as used by existing score analysis [9]. ACE-IQ score categories were determined using WHO-guided question codes and included abuse, neglect, family dysfunction, and violence [10, 11]. The odds ratios (OR) and 95% confidence intervals were estimated from the multiple logistic regression models. A two-sided *P* value of < 0.05 indicates statistical significance.

Data analysis and review were explored by the YPHAs and EIA staff during a third training workshop, following which YPHAs disseminated results to community members, local leaders, national parliamentarians, and the Ministry of Health, as well as within national and international public health and development communities.

## Results

Data were collected from 300 heads of households and 306 adolescents living in the households, 19 schools, and 9 healthcare facilities in three sub-counties in the Lira district of Uganda.

### Household adolescent results

Amongst the surveyed youth, 45.42% were male and 54.58% were female, the majority of which (61.8%) were 14 and 15 years old and enrolled in school (87.5%) (Table 1). The youth primarily resided in temporary grass huts where they shared their sleeping space with other family members and their families relied on subsistence farming from their personal gardens or fields for food. Most of the youth reported having sufficient food to eat, though typical household meal ingredients were starches (84.53%) and beans (95.97%), with lower reported rates of other proteins (7.63%), greens (57.63%), and fruits (19.07%). Most of the respondents listed their mother (64.38%) or father (31.37%) as their primary caretaker. The three most prominent religious belief systems were Roman Catholic (36.93%), Anglican (33.93%), and Pentecostal (27.45%). Sub-county Agweng reported the highest percentage of adolescents not in school (17%), having the lowest percentage of available fruits (23.36%), and the least ability to purchase food at markets (21.51%).

**Table 1:** Household and adolescent demographics

Demographic value	Total	Ayami	Aromo	Agweng
	N (%)	N (%)	N (%)	N (%)
*Age*				
14–15	189 (61.76%)	48 (66.66%)	81 (57.45%)	60 (64.51%)
16–17	117 (38.24%)	24 (33.33%)	60 (42.56%)	33 (35.49%)
*Gender*				
Male	139 (45.42%)	34 (47.22%)	57 (40.43%)	48 (51.61%)
Female	167 (54.58%)	38 (52.78%)	84 (59.57%)	45 (48.39%)
*Attending school*				
Yes	268 (87.58%)	67 (93.06%)	124 (87.94%)	77 (82.80%)
No	38 (12.42%)	5 (6.94%)	17 (12.06%)	16 (17.20%)
*Type of school*				
Government school	230(85.82%)	59 (88.06%)	102 (82.26%)	69 (89.61%)
Private school	36 (13.43%)	6 (8.96%)	22 (17.74%)	8 (10.39%)
Other (e.g. religious/vocational/technical school)	2 (0.74%)	2 (2.98%)	0 (0.00%)	0 (0.00%)
*Housing type*				
Temporary building	148 (49.35%)	82 (55.56%)	71 (48.23%)	68 (46.24%)
Semi-permanent (Mud hut or non-fired brick)	66 (22.22%)	19 (20.83%)	15 (23.40%)	14 (21.51%)
Permanent housing (Fired brick or concrete with iron sheets)	84 (28.10%)	19 (23.61%)	24 (27.66%)	27 (32.26%)
Other	2 (0.33%)	0 (0.00%)	2 (0.71%)	0 (0.00%)
*Number of people sleeping in same room*				
Sleeps alone	85(27.45%)	9 (12.50%)	46 (32.62%)	29 (31.18%)
2 people	130 (42.48%)	39 (54.17%)	50 (35.46%)	41 (44.09%)
3 people	59 (19.28%)	13 (18.06%)	32 (22.70%)	14 (15.05%)
4 or more people	33 (10.78%)	11 (15.28%)	13 (9.22%)	9 (9.68%)
*Primary sources of food*				
Own garden/fields	228 (76.14%)	174 (76.39%)	171 (75.18%)	176 (77.42%)
Purchased at market	70 (23.53%)	16 (23.61%)	17 (24.82%)	15 (21.51%)
Humanitarian aid	2 (0.33%)	0 (0.00%)	0 (0.00%)	1 (1.08%)
*Diet*				
Starch (posho, rice, cassava)	253 (84.53%)	189 (74.77%)	225 (89.12%)	218 86.45%
Protein 1 (beans)	287 (95.97%)	276 (96.40%)	275 (95.92%)	274 (95.79%)
Protein 2 (chicken, beef)	22 (7.63%)	3 (15.32%)	1 (4.08%)	2 (6.07%)
Greens	172 (57.63%)	79 (45.95%)	98 (57.14%)	110 (64.02%)
Fruits	57 (19.07%)	4 (8.11%)	12 (21.09%)	13 (23.36%)
*Primary caretaker*				
Mother	197 (64.38%)	60 (83.33%)	93 (65.96%)	44 (47.31%)
Father	96 (31.37%)	11 (3.59%)	38 (26.95%)	47 (50.54%)
Other (e.g. brother/sister, grandparent, aunt/uncle)	12 (4.25%)	1 (1.39%)	9 (7.09%)	2 (2.16%)
*Household religion*				
Anglican	104 (33.99%)	29 (40.28%)	49 (34.75%)	26 (27.96%)
Roman Catholic	113 (36.93%)	12 (16.67%)	55 (39.01%)	46 (49.46%)
Pentecostal	84 (27.45%)	38 (38.89%)	37 (26.24%)	19 (20.43%)
Muslim	3 (0.98%)	3 (4.17%)	0 (0.00%)	0 (0.00%)

### Self-reported health behaviors and outcomes results

Most youth (> 85%) indicated they were in good physical health, though households reported high rates of malaria and diarrhea (Table 2). Over 10% of youth, with percentages higher in male adolescents than females, reported having never seen a doctor (an indicator of neglect) and 50% indicated that they had been denied care at a healthcare facility. Most youths spent substantial time (> 5 h a day) collecting water and cooking, with girls reporting more time spent on these activities than boys. While the adolescents reported low personal substance use, nearly all adolescents believed that at least some of their friends use tobacco, alcohol, or other drugs and most (87.3%) believed that the harmful use of alcohol is a problem in their community. Over 20% indicated that their parent/guardian sometimes or often drank too much and became abusive at home. General health, care at health facilities, household tasks, and substance use were statistically significantly associated with gender.

**Table 2:** Genderwise comparison of adolescent self-reported health behaviors and health outcomes

	Total	Male	Female	*P* value*
	N (%)	N (%)	N (%)	
*Physical health*				
General health				
Excellent	69 (22.55%)	40 (28.78)	29 (17.37%)	**0.06**
Good	194 (63.40%)	79 (56.83%)	115 (68.86%)	
Fair	33 (10.78%)	14 (10.07%)	19 (11.38%)	
Poor	10 (3.27%)	6 (4.32%)	4 (2.4%)	
Last visit with medical professional				
In the past year	239 (78.10%)	100 (71.94%)	139 (83.23%)	**0.03**
3–5 years ago	27 (8.82%)	19 (13.67%)	8 (4.79%)	
> 10 years ago	5 (1.63%)	3 (2.16%)	2 (1.20%)	
Never	35 (11.44%)	17 (12.23%)	18 (10.78%)	
Denied care from a health center				
Yes	154 (50.33%)	81 (58.27%)	73 (43.71%)	**0.01**
No	152 (49.67%)	58 (41.73%)	94 (56.29%)	
Illness in the past month (diarrhea)				
Yes	91 (29.74%)	42 (30.22%)	49 (29.34%)	0.87
No	215 (70.26%)	97 (69.78%)	118 (70.66%)	
Weekly time spent preparing meals for household				
0 h	20 (6.54%)	20 (14.39%)	0 (0.00%)	**0.00**
1–2 h	72 (23.53%)	32 (23.02%)	40 (23.95%)	
3–4 h	86 (28.10%)	47 (33.81%)	39 (23.35%)	
> 5 h	128 (41.83%)	40 (28.78%)	88 (52.69%)	
Weekly time spent collecting water for household				
0 h	65 (21.24%)	29 (20.86%)	36 (21.56%)	**0.00**
1–2 h	69 (22.55%)	43 (30.94%)	26 (15.57%)	
3–4 h	46 (15.03%)	24 (17.27%)	22 (13.17%)	
> 5 h	126 (41.18%)	43 (30.94%)	83 (49.70%)	
*Substance use*				
Tried tobacco products				
Yes	13 (3.92%)	5 (3.60%)	7 (4.19%)	0,79
No	294 (96.08%)	134 (96.4%)	160 (95.81%)	
Tried alcohol				
Yes	38 (12.42%)	23 (16.55%)	15 (8.98%)	**0.07**
No	268 (84.97%0	116 (83.45%)	152 (91.01%)	
Alcohol use in the community				
Too many people drink too much	267 (87.25%)	127 (91.37%)	140 (83.83%)	0.233
A few people drink too much	23 (7.52%)	6 (4.31%)	17 (10.17%)	
People in my community do not drink too much	16 (5.22%)	6 (4.31%)	10 (5.99%)	
Substance use by friends (tobacco, drugs)				
All my friends use substances	3 (0.98%)	0 (0.00%)	3 (1.80%)	**0.00**
Most of my friends use substances	14 (4.58%)	11 (7.91%)	3 (1.80%)	
Some of my friends use substances	289 (94.44%)	128 (92.09%)	161 (96.41%)	
*Safety*				
Experienced violence in home				
Often	67 (21.90%)	27 (19.42%)	40 (23.95%)	0.45
Sometimes	239 (78.10%)	112 (80.57%)	127 (76.04%)	

*P < 0.05 was considered significant using Pearson Chi Square Test

### Adolescent social support and mental health belief results

Adolescents generally reported having adequate social support with differences between sub-counties (Table 3). Seventy-five percent of adolescents reported speaking to their mother when having worries or concerns about mental health, with adolescents in Agweng sub-county being least likely to be able to talk to an adult when needed. The COVID-19 pandemic significantly impacted adolescent social support, with 37% of adolescents experiencing feelings of sadness and anxiousness and 60% of adolescents experiencing social isolation during lockdowns. Adolescents primarily reported believing mental health problems were caused by witchcraft (29%) and God (14%) with strong differences in beliefs between sub-counties.

**Table 3:** Adolescent social support and mental health beliefs

	Total	Ayami	Aromo	Agweng
	N (%)	N (%)	N (%)	N (%)
*Social support*				
I can ask adults for help when I need it				
Often	113 (36.93%)	22 (30.56%)	58 (41.13%)	33 (35.48%)
Sometimes	115 (37.58%)	36 (50%)	43 (30.50%)	36 (38.71%)
Rarely	60 (19.61%)	13 (18.06%)	25 (17.73%)	22 (23.66%)
Never	18 (5.88%)	1 (1.39%)	15 (10.64%)	2 (2.15%)
I know where to go if I or someone I know is suffering from mental Illness				
Yes	184 (60%)	35 (48.61%)	72 (51.06%)	77 (82.80%)
No	122 (40%)	37 (51.39%)	69 (48.94%)	16 (17.20%)
How many close friends do you have				
0	7 (2.29%)	2 (2.78%)	5 (3.55%)	0 (0.00%)
1–2	83 (27.12%)	15 (20.83%)	42 (29.79%)	26 (27.96%)
3–4	109 (35.62%)	29 (40.28%)	51 (36.17%)	29 (31.18%)
> 5	107 (34.97%)	26 (36.11%)	43 (30.50%)	38 (40.86%)
Who do you usually talk to if you have worries or concerns				
Mother	230 (75.16%)	61 (84.72%)	105 (74.47%)	64 (68.82%)
Father	46 (15.03%)	10 (13.89%)	16 (11.35%)	20 (21.51%)
Brother/sister	8 (2.61%)	0 (0.00%)	3 (2.13%)	5 (5.38%)
Friends/peers	8 (2.61%)	0 (0.00%)	7 (4.96%)	1 (1.08%)
Grandparent or other family member	7 (2.28%)	0 (0.00%)	4 (2.84%)	1 (1.08%)
I do not have anyone to speak to	7 (2.29%)	6 (1.39%)	1 (0.71%)	0.00%
*Impacts of COVID-19 lockdowns on social support*				
Experienced feelings of extreme Sadness/anxiousness	113 (37%)	18 (16.03%)	67 (59.43%)	27 (24.52%)
Experienced social isolation (loss of contact with friends)	183 (60%)	28 (15.64%)	93 (50.83%)	61 (33.52%)
*Mental health beliefs*				
Mental health problems are caused by				
Witchcraft	89 (29.31%)	6 (7.14%)	48 (55%)	35 (29.55%)
God’s will	43 (14.37%)	4 (11.43%)	3 (8.33%)	36 (27.27%)
Chemicals/hormone imbalances	42 (13.79%)	33 (25.71%)	9 (10%)	0 (0.00%)
Traumatic experiences	24 (8.05%)	1 (4.29%)	3 (6.67%)	20 (15.91%)
Family history	35 (11.49%)	5 (12.86%)	2 (6.67%)	5 (15.91%)
Don’t know	70 (22.99%)	26 (38.57%)	9 (13.33%)	7 (11.36%)

### WHO-5 well-being scale results

**Fig. 1:**
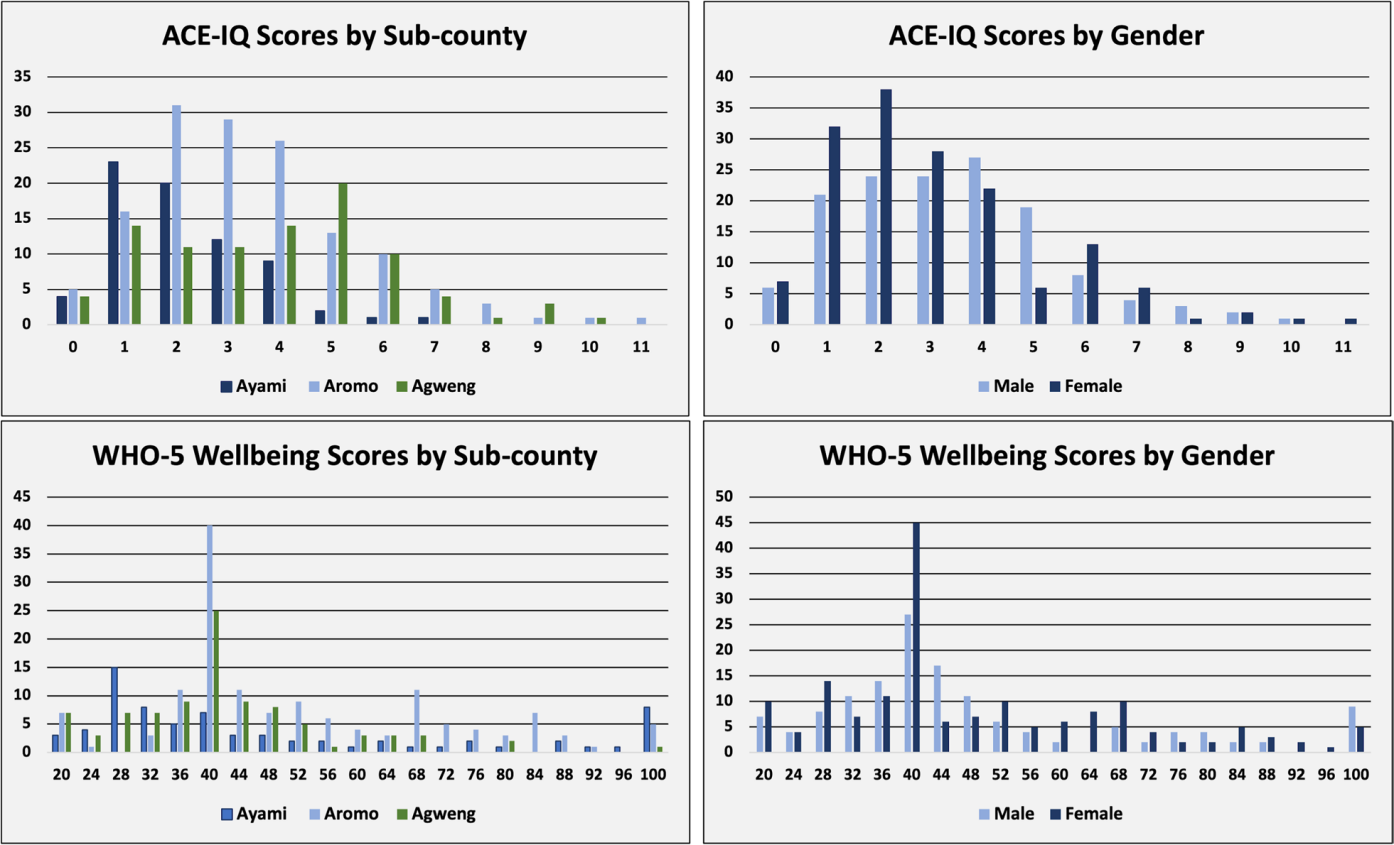
ACE-IQ and WHO-5 adolescent well-being scores by sub-county and gender

On the WHO-5 Well-being scale, scores ranged from 20 to 100 (Fig. 1), with significant variation between sub-counties and gender. Two-hundred and three of the 306 adolescents surveyed, or 66% of adolescents, scored lower than 52 points, indicating poor well-being and possible symptoms of depression amongst the surveyed youth. Scores of 28 or below, scored by 15% of surveyed youth, are considered indicative of clinical depression. The average adolescent well-being score was 48 points. Adolescents in Agweng sub-county, the most rural and low-income community surveyed, scored the lowest with 96.77% of respondents falling below a score of 75. Adolescent females scored lower than adolescent males, with only 11% of females scoring higher than 75. Adolescents with 5 or more friends were 79% less likely to report experiencing depressive symptoms. The number of close friends was found to be a statistically significant predictor of reporting depressive symptoms on the WHO-5 Index (*p *< 0.05) (Table 4). Knowledge of where to seek help when struggling with mental illness was also a significant protective factor for adolescent depression, with adolescents reporting they know where to go when facing mental illness 45% less likely to report symptoms of depression.

**Table 4:** Multivariate logistic regression model for relationship between social support, adverse childhood experience score categories, and WHO-5 index depressive symptoms

	OR (CI)	*P* value**
*Social support*		
Can ask adults for help when needed	0.71 (0.28,1.76)	0.454
Knows where to go if suffering from mental Illness	0.55 (0.31,0.99)	**0.04**
Number of close friends		
1–2	0.23 (0.036,1.53)	0.13
3–4	0.35 (0.06,2.14)	0.26
> 5	0.21 (0.03,1.33)	**0.098**
*Adverse childhood experiences*		
Abuse	0.99 (0.62,1.10)	0.62
Neglect	0.88 (0.51,1.50)	0.63
Family dysfunction	0.61 (0.57,1.13)	**0.01**
Violence	1.04 (0.69,1.56)	0.88

**P < 0.05 was considered significant

### Adverse childhood experience score results

Adverse Childhood Experience (ACE-IQ) scores for adolescents ranged from 0 to 11, with an average score of 3 out of 13 (Fig. 1). Forty-one percent of adolescents had ACE-IQ scores of 4 or higher. In all 3 sub-counties, females scored higher than males, with females in Agweng scoring the highest on the ACE-IQ survey tool indicating having experienced the most adverse childhood experiences. ACE-IQ scores for adolescent family dysfunction were statistically significantly associated with reporting depressive symptoms on the WHO-5 index (*p *< 0.05) (Table 4). Adolescents reporting family dysfunction scores lower than 5 were 39% less likely to report experiencing symptoms of depression.

### School and health facility results

Data was collected from 19 schools (16 public and 3 private) and 9 health facilities (4 health center IIs, 2 health center IIIs, and 3 specialty care centers). Fifteen schools, 13 of which were government schools, reported having mental health resources available for students, including student safety guidelines, counselors, and emergency response services. Twelve schools reported having activities or events related to mental health, and only 10%, 2 schools, reported including mental health information in their curriculum. Eighty-eight percent, or 8 health facilities, indicated that they do not provide mental health services, though 5 facilities reported having a trained mental health professional on staff. Seventy-seven percent of health facilities reported having safe spaces available to provide confidential services to adolescents.

## Discussion

The study results indicate that adolescents throughout the Lira District in northern Uganda suffer from poor mental health, limited access to mental health services, and persistent myths that lead to stigma related to mental health conditions. Moreover, the study results point to multiple politically relevant issues linked to mental health outcomes.

Many adolescents surveyed exhibited poor mental health status. The majority scored in the lowest percentile of well-being, indicating that further screening and treatment for depression is clinically recommended [9]. Nearly half of the adolescents surveyed reported ACE-IQ scores of 4 or higher which has been linked to a doubled risk of stroke or heart disease, a tripled risk of chronic depression or alcoholism, and an increased risk of being a victim of violence. [11]. The study also took place just as the COVID pandemic lockdowns were coming to an end and survey respondents reported significant impacts of the lockdowns on social isolation and wellness. Findings are consistent with previous literature noting significant increases in feelings of loneliness, psychological distress, and fear among adolescents throughout COVID lockdowns [12].

Notably, girls were more likely to report higher instances of abuse, neglect, family dysfunction, and violence. Our results are consistent with other studies that have found consistently higher rates of gender-based violence in sub-Saharan Africa than the global average, with previous literature citing more than two in five women have experienced some circumstance of violence [13]. Recent studies have also shown a noticeable and disproportionate increase in the global burden of depressive and anxiety disorders during COVID among women and girls worldwide [2]. Girls in Lira were similarly likelier to report being sad and anxious and losing friends during the pandemic. There are multiple reasons for this unequal impact. For example, adolescent girls in this study indicated having spent more time than males performing household tasks such as food preparation and fetching water and reported having fewer close friends. Societal gender roles and norms, including taking on more housework, were likely stressed during COVID when girls lacked the physical and social breaks offered by school and potentially further limited girls’ ability to engage in social activities important for good mental health outcomes and well-being.

Sub-county was a statistically significant factor impacting both wellness and adverse childhood event outcomes. Most of the adolescents in the study, regardless of sub-county, lived in poverty as indicated by their housing type and source of food. Many indicated exposure to domestic violence in their homes and a substantial percentage reported having never seen a doctor, an accepted indicator of neglect [9]. However, Agweng had the highest percentages of all the above, recorded poorer access to services as compared to Aromo and Ayami, and reported the worst outcomes on the WHO-5 Index and ACE-IQ scales. These findings are consistent with existing literature, linking household and community poverty and accessibility to poorer mental health outcomes [14].

Although few households in the region were not impacted by the decades-long turmoil, Agweng was ground zero during the LRA conflict. This could also partially explain the poorer outcomes in the sub-county. The adolescents surveyed have no living memory of the war, however, they do have memories of the immediate aftermath, have grown up in communities struggling to rebuild post-conflict, and have been raised by parents who likely suffered directly. The high levels of verbal abuse, physical abuse, neglect, and family dysfunction reported are likely in part due to trauma-induced mental health conditions among parents and other caretakers. Poor adolescent wellness scores could also reflect intergenerational trauma, which also places them at increased risk for mental illness and other chronic diseases [15].

Unfortunately, despite the high prevalence of poor mental health indicators, the adolescents surveyed lacked general awareness about the causes and effects of mental illness, with a high percentage of adolescents attributing mental health problems to God and witchcraft. Moreover, survey results from schools indicated that little information about mental health was being formally taught to adolescents in the region. The lack of awareness about the relationship between trauma and mental illness is troubling given the burden of intense violence and war experienced within the communities. Alternatively, the dominance of religious or spiritual explanations for mental illness and the use of spiritual healers for treatment can lead to stigma around conditions and reluctance to seek help through the formal health system. Although often prioritized in high-income countries, little attention has been paid to address stigma in lower-income countries where religiously reinforced stigma is often more common and gaps in mental health treatment are larger [16]. Collaboration and increased education amongst care providers, spiritual and religious healers, educators, and adolescents are important for confronting community-level factors influencing mental health outcomes.

The risk factors identified in this study, including poverty, gender, conflict-derived trauma, stigma, and lack of treatment resources point to the need for political examinations and solutions to the burden of mental illness. This study once again shows that poor mental health disproportionately impacts the weakest and most marginalized in society; namely poor, rural, women and girls. The data also indicate a lack of policy and government intervention and investment in mental health at the local level as evidenced by the low capacity of schools and health facilities. It is a reinforcing loop, in which those most impacted lack representation and access to positions of power, which constrains their ability to advocate for their needs. This lack of agency and influence perpetuates and reinforces their real and perceived feelings of vulnerability, further increasing their risk of poor mental health. Alternatively, programs that focus on shifting power structures and the political economy in communities and give voice to those most at risk are likely to positively impact community wellness and mental health.

Study limitations include that households were selected by convenience, and thus may not represent all households or adolescents in the region. The data were also collected and analyzed by novice youth researchers who may have made mistakes throughout the process, though the results were largely consistent with existing literature. Youth involvement in the study can also be viewed as a strength as they were potentially able to elicit honest answers from their peers as opposed to older data collectors whom the local youth may not trust. Moreover, traditional approaches to studying mental health in Uganda and in other low-income countries are often linked to historically colonist-enforced biological and ecological methods that lack local community perspectives and engagement [17]. Past international evaluations of mental health have focused on the importance of ‘closing the treatment gap’ using Western diagnostic tools and interventions and have failed to address persistent societal determinants, cultural myths, and traditional practices [18]. The CBPR method used in this study, alternatively, sought to build on community strengths by democratizing the research process and empowering young leaders to collect and analyze their community’s attitudes and behaviors and use the information to educate their community members about the mental health challenges they face, and to advocate to local, regional, and national stakeholders for more resources for mental health services.

## Conclusion

This study collected data on mental health and wellness status, knowledge, beliefs, and behaviors amongst adolescents living in households located in three sub-counties in Lira District, Uganda heavily impacted by war. The study results provide useful insight into the impact of the conflict on the health and wellness of the subsequent generation of youth in the region. It also highlights the need for politically informed analyses and interventions to adequately address the causes of poor mental health in Uganda and beyond. The data collection methods also provide a unique model of how mental health-related research and programming can be carried out in a decolonized manner with the potential to make lasting impacts on community health, including mental health, many times greater than previous research models.

## Data Availability

The datasets used and/or analyzed during the current study are available from the corresponding author upon reasonable request.

## Abbreviations

WHO: World Health Organization
CBPR: Community-Based Participatory Research
LRA: Lord’s Resistance Army
USC: University of Southern California
YPHA: Youth Public Health Ambassadors
CCI: Children’s Chance International
ACE-IQ: Adverse Childhood Experiences Score
